# Mutagenicity, cytotoxic and antioxidant activities of *Ricinus communis* different parts

**DOI:** 10.1186/s13065-018-0370-0

**Published:** 2018-01-19

**Authors:** Mazhar Abbas, Abid Ali, Muhammad Arshad, Asia Atta, Zahed Mehmood, Imtiaz Mahmood Tahir, Munawar Iqbal

**Affiliations:** 1 Department of Basic Sciences, Section Biochemistry, College of Veterinary and Animal Sciences, Jhang Campus, Jhang, 35200 Pakistan; 2 College of Allied Health Professional, Directorate of Medical Science, Govt. College University, Faisalabad, Pakistan; 3 Department of Biochemistry, Bahauddin Zakariya University, Multan, 60800 Pakistan; 4 Department of Applied Chemistry and Biochemistry, Govt. College University, Faisalabad, Pakistan; 5 Department of Chemistry, The University of Lahore, Lahore, Pakistan

**Keywords:** Medicinal plant, Extraction techniques, Antioxidant, DNA induced damage, Bioassays

## Abstract

*Ricinus communis* (castor plant) is a potent medicinal plant, which is commonly used in the treatment of various ailments. The present study was conducted to appraise the cytotoxicity and mutagenicity of *R. communis* along with antioxidant and antimicrobial activities. Cytotoxicity was evaluated by hemolytic and brine shrimp assays, whereas Ames test (TA98 and TA100) was used for mutagenicity evaluation. Plant different parts were extracted in methanol by shaking, sonication and Soxhlet extraction methods. The *R. communis* methanolic extracts showed promising antioxidant activity evaluated as through total phenolic contents (TPC), total flavonoid content (TFC), DPPH free radical inhibition, reducing power and inhibition of linoleic acid oxidation. *R. communis* seeds, stem, leaves, fruit and root methanolic extracts showed mild to moderate cytotoxicity against red blood cells (RBCs) of human and bovine. Brine shrimp lethality also revealed the cytotoxic nature of extracts with LC_50_ in the range of 0.22–3.70 (µg/mL) (shaking), 1.59–60.92 (µg/mL) (sonication) and 0.72–33.60 (µg/mL) (Soxhlet), whereas LC_90_ values were in the range of 345.42–1695.81, 660.50–14,794.40 and 641.62–15,047.80 µg/mL for shaking, sonication and Soxhlet extraction methods, respectively. *R. communis* methanolic extracts revealed mild mutagenicity against TA98 (range 1975 ± 67 to 2628 ± 79 revertant colonies) and TA100 (range 2773 ± 92 to 3461 ± 147 revertant colonies) strains and these values were 3267 ± 278 and 4720 ± 346 revertant colonies in case of TA98 and TA100 positive controls, respectively. *R. communis* methanolic extracts prevented the H_2_O_2_ and UV to Plasmid pBR^322^ DNA oxidative damage. Results revealed that *R. communis* is a potential source of bioactive compounds and in future studies the bioactive compounds will be identified by advanced spectroscopic techniques. 
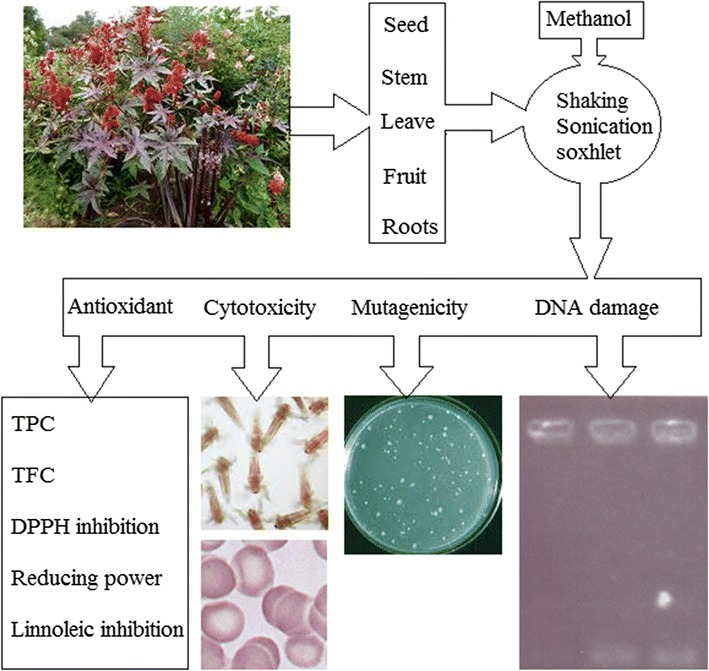

## Introduction

Medicinal plants are commonly used to treat various ailments in most of the developing communities. Besides, these are a potent source of food, fodder and fuel, etc. Ethnopharmacology involves the investigation of those plants used by traditional communities without understanding the pharmacological basis of medicinal plants [1–3]. *Ricinus communis* (family *Euphorbiaceae*) is a soft wood small tree, located in tropical and warm temperate regions of the world and bioactivity has been studied well of this plant [4, 5]. *R. communis* plant is used for the treatment of hepatitis, skin and breast cancer [6]. Naturally, plants synthesize phytochemicals as a part of their defense system under variable and harsh environmental conditions, which provide defense for plants against microorganism, pests and insects [7–13]. In developing country, plant derived herbal medicine are used commonly due to easy access and affordable, which are also regarded as safe versus synthetic drugs [14–17]. Moreover, it is believed that plant based bioactive compounds have no side effects as compared to synthetic drugs and has wide range of therapeutic applications [18, 19]. However, plant extracts may contain toxic compounds [20], which can harm the living organisms. *R. communis* seeds, leaves, fruit, stem and bark are used in different traditional therapeutic practices by local practitioner (Hakeem) [21]. Therefore, the toxicity profiling (using bioassays) of such important plants is very helpful to appraise the safety [22–33]. In this regard, the bioassays (hemolytic and brine shrimp) are the standard tests to evaluate the cytotoxicity, whereas TA98 and TA100 (based on *salmonella* mutant strains) are the reference tests for mutagenicity evaluation. The shrimp lethality assay was developed by Michael [34], later Vanhaecke [35], and Sleet and Brendel [36]. In this assay, *Artemia* nauplii are exposed to test compound and lethality is used to estimate cytotoxicity. This has been used as a useful tool for preliminary assessment of toxicity [37] i.e., fungal [38], extract [39, 40], metals [41], toxins  [42], pesticides [43], wastewater [44–48], fumonisins [49] and dental materials [50]. Various authors also utilized this hemolytic test for cytotoxicity evaluation of different systems [51–56]. The Ames test was proposed by Ames and coworker [57–59] and have been used for mutagenicity evaluation of tobacco smoke [60], wastewater [61], treated wastewater [44, 45], herbal extracts [62] and toxic chemicals [63].

In view of importance of *R. communis* as a medicinal plant, nevertheless, researcher focused on cytotoxicity and mutagenicity using standard assays. Therefore, the principal objectives of the present study were to investigate the cytotoxicity and mutagenicity of different parts of *R. communis* parts along with bioactivity profiling. Hydrogen peroxide induced DNA damage protective efficiency was also evaluated of the extracts.

## Materials and methods

### Plant material

*Ricinus communis* plant was collected from the Botanical Garden, University of Agriculture, Faisalabad, Pakistan and seeds were purchased from local market, Faisalabad. The plants and seeds specimens were identified by Botanist, Dr. Mansoor Hameed, Department of Botany University of Agriculture Faisalabad, Pakistan.

### Sample preparation and extraction

The collected leaves, stem, fruit, roots and seeds of *R. communis* were washed with distilled water and shade dried. Dried plant parts were ground and passed through 80 mm mesh size. Different parts (20 g) were extracted in methanol (100 mL) using shaking, Soxhlet and sonication extraction methods. In case of shaking, extraction was performed for 6 h at room temperature (Shaker Gallenkamp, UK). For sonication, ultrasonic treatment (42 kHz, 135 W; Branson ultrasonic corporation, USA) was applied for 30 min. For Soxhlet, extraction was performed in Soxhlet extractor for 3 h. After extraction, methanol was evaporated and concentrated extracts were stored at − 4 °C.

### Antioxidant activity

#### Total phenolic contents (TPC)

The TPC was assessed using Folin–Ciocalteu reagent following reported method elsewhere [64]. The TPC was calculated using a calibration curve (gallic acid, 10–100 ppm) and data was expressed as GAE of dry plant matter.

#### Total flavonoid contents (TFC)

Extract (0.1 g/mL) was placed in 10 mL volumetric flask and 5 mL distilled water was added. Then, 0.3 mL of 5% NaNO_2_ was added and after 5 min, 0.6 mL of 10% AlCl_3_ was added. After another 5 min, 2 mL of 1 M NaOH was added, mixed well and absorbance was measured at 510 nm. TFC amount was evaluated as catechin equivalents (g/100 g of DM) [65].

#### DPPH Radical scavenging assay

For DPPH activity measurement, extract (0.1 mg/mL) were mixed with 1 mL of 90 µM DPPH solution and then, final volume was made to 4 mL by adding 95% methanol. After 1 h of incubation at room temperature, the absorbance was recorded at 515 nm and response was calculated as in Eq. 1 [66].

1 $$Inhibition \, ( \%) = \left[ {\frac{{A_{0} }}{{A_{s} - A_{0} }}} \right]*100$$

where, *A*_o_ is the absorbance of the control and *A*_*s*_ is the absorbance of the extract (sample).

#### Antioxidant activity in linoleic acid system

The percent inhibition of peroxidation of linoleic acid system [67]. Extract (5 mg) and linoleic acid (0.13 mL), 99.8% ethanol (10 mL) and 10 mL of 0.2 M sodium phosphate buffer (PH 7.0) were mixed thoroughly. Then, 25 mL with distilled water was added and incubated at 40 °C. The degree of oxidation was measured following thiocyanate method and percent inhibition of linoleic acid was calculated using Eq. 2.

2 $$Inhibition \, ( \%) = 100 - \left[ {\frac{{A_{{s,175\, {\text{h}}}} }}{{A_{{0, 175 \,{\text{h}}}} }}} \right]*100$$

where, A_s,175 h_ and A_0,175 h_ are the absorbance values at 175 h of sample and control, respectively.

#### Reducing power determination

The reducing power was determined as described elsewhere [68]. Sodium phosphate buffer (5.0 mL, 0.2 M, pH 6.6), and potassium ferricyanide (5.0 mL, 1.0%) and *R. communis* extract was mixed and incubated at 50 °C for 20 min. Then, 5 mL of trichloroacetic acid (10%) was added and centrifuged at 980*×g* for 10 min at 5 °C. The supernatant (5.0 mL) was collected and diluted with distilled water (5.0 mL) along with ferric chloride (1.0 mL, 0.1%) addition and absorbance was recorded at 700 nm (Hitachi U-2001, Tokyo, Japan).

### Toxicity evaluation

#### Hemolytic assay

Powell [69] method was adopted for hemolytic test. Blood sample (human and bovine, collected in heparinized tubes) was centrifuged for 5 min at 850×*g* for three to five times using chilled (4 °C) sterile isotonic phosphate buffer saline (PBS) having pH 7.4 and RBCs were separated. The separated RBCs were suspended in the PBS. Erythrocytes were counted using hemocytometer, which were 7.068 × 10^8^ cells/mL. Then, 20 µL of plant extract was mixed with 180 µL blood cell suspension and samples were incubated with agitation for 30 min at 37 °C. The tubes were placed on ice for 5 min and contents were centrifuged for 5 min at 1310×*g*. A 100 µL supernatant was taken and 900 µL chilled PBS was added and eppendorfs were placed on ice for 5 min and absorbance was noted at 576 nm (BioTek, Winooski, VT, USA). The RBCs lysis (%) was calculated using relation shown in Eq. 3.

3 $$RBC_{lysis(\% )} = \left[ {\left( {{{A_{s} } \mathord{\left/ {\vphantom {{A_{s} } {A_{tx - 100} }}} \right. \kern-0pt} {A_{tx - 100} }}} \right) \times 100} \right]$$

where *A*_s_ is absorbance of the sample and *A*_tx−100_ is the absorbance of Triton X-100. Triton X-100 (0.1%) was used as a positive control and PBS was used as negative control.

#### Brine shrimp lethality assay

Brine shrimp (*Artemia* sp.) eggs were hatched in a culture flask (15 × 15 × 15 cm) filled with sterile, artificial seawater (prepared using sea salt 38 g/L, the pH was adjusted to 8.5 with 1 M NaOH) under constant aeration (aquarium air pump) and illumination for 48 h at 25 °C. After 48 h the shrimp-larvae were collected and exposed extract under investigation. The brine shrimp lethality assay was performed following reported method [39, 70]. Plant extracts were diluted to concentrations of 10, 100, 1000 and 3000 µg/mL for cytotoxicity testing. Twenty brine shrimp larvae were placed in vials containing extract using a plastic pipette with a 2 mm diameter tip. The larvae survival was counted under the stereomicroscope after 24 h and percent death rate at each dose and control were calculated. Salt-water and cyclophosphamide were used as negative and positive controls, respectively, and LC_50_ and LC_90_ values were estimated.

#### Ames test

Two *S. typhimurium* strains TA98 and TA100 were used [71]. The extract was considered mutagenic, if the number of revertant colonies on the plates containing test compounds was twice the number of revertant colonies in control plates (background) (extract/control revertant colonies ≥ 2.0) [72]. All the experiments were performed in triplicates and data, thus obtained was expressed as mean ± SD.

## Results and discussion

### Antioxidant activity

The antioxidant activity results are shown in Table 1. It was observed that extraction methods showed variable antioxidant activities in spite of same plant parts were used, however, all plant parts furnished promising antioxidant activities. The sonication extraction method showed higher TPC followed by Soxhlet and shaking and a similar trend was observed in case of TFC, DPPH percentage inhibition, reducing power and linoleic acid inhibition. The TPC, TFC, DPPH percentage inhibition, reducing power and linoleic acid inhibition values in case of sonication (for seeds) were 361 ± 2 (mg/100 g), 171 ± 2.8 (mg/100 g), 8.8 ± 0.6 (%), 87.28 ± 0.1 (%) and 0.854 ± 0.3 (OD), whereas Soxhlet showed these values 149 ± 1.5 (mg/100 g), 94 ± 0.4 (mg/100 g), 7.42 ± 0.5 (%), 48.19 ± 0.3 (%) and 0.523 ± 0.7 (OD) and in case of shaking 122 ± 3 (mg/100 g), 15 ± 1 (mg/100 g), 7.25 ± 0.3 (%), 43.56 ± 0.3 (%) and 0.481 ± 0.8 (OD) were recorded, respectively. The antioxidant in case of extraction methods and among plant parts found significantly different (*P* < 0.05). in case of shaking extraction method, leaves showed higher TPC and TFC values followed by seed, fruit, stem and roots, whereas in case of DPPH the trend was as; stem > leaves > seeds > roots > fruit. The reducing power of plant parts extracts was found in following order; leaves > seeds > fruits > stem and roots and linoleic acid percentage inhibition was found in following order; leaves > seeds > fruit > stem > roots. The antioxidant activity trend for different parts for sonication and Soxhlet also showed same trend, i.e., in case of sonication, the TFC values were recorded to be 361 ± 2, 11 ± 0.3, 58 ± 1, 64 ± 2 and 12 ± 0.5 (mg/100 g), TFC values were 171 ± 2.8, 4 ± 0.6, 32 ± 1.2, 46 ± 1.2 and 2.8 ± 0.6 (mg/100 g) and 8.8 ± 0.6, 6.2 ± 0.9, 10.45 ± 0.7, 5.67 ± 0.1 and 13.29 ± 0.7 (%) of DPPH percentage inhibition for seeds, stem, leaves, fruit and roots. The reducing power of seeds, stem, leaves, fruits and roots were 87.28 ± 0.1, 8.14 ± 0.7, 20.64 ± 0.3, 23.54 ± 0.6 and 11.39 ± 0.2 (%) and linoleic acid percentage inhibition values were recorded to be 0.854 ± 0.3, 0.184 ± 0.2, 0.356 ± 0.8, 0.379 ± 0.3 and 0.234 ± 0.9 (OD) for seeds, stem, leaves, fruits and root extracts, respectively. Earlier, it is also reported that the aerial part of *R. communis* has potent antioxidant activity [73] and in present investigation, leaves and seeds showed considerable higher (*P* < 0.05) higher antioxidant activity versus other parts. Antioxidant activity of *n*-hexane, dichloromethane, acetone, and methanol extracts of *R. communis* was also quantified using ABTS^+^ method. Among all extracting solvents, methanol extract showed the highest percentage free radical scavenging activity (95%) followed by acetone (91%), dichloromethane (62%), and *n*-hexane (50%). The antioxidant activity of *R. communis* seeds have also been reported previously [74] and antioxidant activity was comparable with present investigation. Nevertheless, the comparative studies based on different parts using different extraction methods were performed. So far, present investigation indicates that *R. communis* different parts had promising antioxidant activities; however, antioxidant activities were variable depending upon plant parts and extracting methods.

**Table 1:** Antioxidant profile of extracts of *Ricinus communis* different parts, extracted by different extraction methods

S. No.	Method	Plants part	TPC (mg/100 g)	TFC (mg/100 g)	DPPH inhibition (%) (0.1 mg/mL)	Linoleic acid inhibition (%)	R. Power (1 mg/mL) (OD)
1	Shaking^C^	Seed^b^	122 ± 3	15 ± 1	7.25 ± 0.3	43.56 ± 0.3	0.481 ± 0.8
2		Stem^d^	24 ± 1	6 ± 0.2	20 ± 0.2	12.46 ± 0.7	0.278 ± 0.3
3		Leave^a^	165 ± 1.5	71 ± 1	7.54 ± 0.2	57.38 ± 0.2	0.578 ± 0.6
4		Fruit^c^	94 ± 2	68 ± 2	5.14 ± 0.3	35.69 ± 0.4	0.396 ± 0.1
5		Root^e^	16 ± 1	4 ± 0.1	6.58 ± 0.8	10.84 ± 0.9	0.209 ± 0.7
6	Sonication^A^	Seed^a^	361 ± 2	171 ± 2.8	8.8 ± 0.6	87.28 ± 0.1	0.854 ± 0.3
7		Stem^c^	11 ± 0.3	4 ± 0.6	6.2 ± 0.9	8.14 ± 0.7	0.184 ± 0.2
8		Leave^b^	58 ± 1	32 ± 1.2	10.45 ± 0.7	20.64 ± 0.3	0.356 ± 0.8
9		Fruit^b^	64 ± 2	46 ± 1.2	5.67 ± 0.1	23.54 ± 0.6	0.379 ± 0.3
10		Root^c^	12 ± 0.5	2.8 ± 0.6	13.29 ± 0.7	11.39 ± 0.2	0.234 ± 0.9
11	Soxhlet^B^	Seed^a^	149 ± 1.5	94 ± 0.4	7.42 ± 0.5	48.19 ± 0.3	0.523 ± 0.7
12		Stem^c^	5 ± 0.1	16 ± 0.1	14.33 ± 0.9	6.63 ± 0.5	0.194 ± 0.4
13		Leave^b^	31 ± 1	39 ± 0.6	13.99 ± 0.4	26.32 ± 0.6	0.376 ± 0.6
14		Fruit^b^	23 ± 0.5	34 ± 0.3	6.9 ± 0.8	21.21 ± 0.9	0.362 ± 0.2
15		Root^c^	9 ± 0.9	2 ± 0.1	8.24 ± 0.6	7.23 ± 0.3	0.231 ± 0.8

The values are the mean ± SD of triplicate experiments. Capital letters in superscripts are representing significant different among extraction methods (*P* < 0.05) and small letter in superscripts are representing significance difference (*P* < 0.05) in activity within plant parts for individual extraction methods

### Toxicity

The cytotoxicity of *R. communis* different methanolic extract was evaluated through hemolytic and brine shrimp assays. The hemolytic activity of the extracts was compared with Triton X-100 (positive control-100% RBCs lysis) and PBS (negative control-0% lysis). The lysis results of both human and bovine RBCs are shown in Table 2. In case of shaking, *R. communis* methanolic extracts showed cytotoxicity in the range of 3.51–50.9% (human RBCs % lysis) and 2.23–44.91% (bovine RBCs % lysis), whereas sonication revealed the cytotoxicity in the range of 0.76–15.56% (human RBCs) and 0.71–13.32% (bovine RBCs) and in case of Soxhlet method, the human RBCs and bovine RBCs lysis percentages were 0.70–34.20% and 0.07–41%, respectively. The *R. communis* plant parts also showed different cytotoxic effects and in case of In the case of human RBCs, the cytotoxicity was in following order; seeds > fruits > leaves > roots > stem (shaking), leaves > roots > fruits > seeds > stem (sonication) and leaves > fruits > stem > roots > seed (Soxhlet). Similar trend was observed in case of bovine RBCs lysis, however, *R. communis* all parts showed slightly less RBCs lysis in case of bovine RBCs versus human RBCs.

**Table 2:** Human and bovine red blood cell lysis (RBCs) assays of *Ricinus communis* different parts, extracted by different extraction methods

S. No.	Method	*R. communis*	Human RBC	Bovine RBC
1	Shaking^A^	Seed^a^	50.91 ± 1.32	44.91 ± 0.34
2		Stem^e^	3.51 ± 0.63	2.23 ± 0.08
3		Leave^c^	28.22 ± 0.27	26.58 ± 0.07
4		Fruit^b^	38.48 ± 0.37	37.34 ± 0.26
5		Root^d^	12.62 ± 0.23	8.30 ± 0.51
6	Sonication^B^	Seed^b^	6.55 ± 0.19	5.55 ± 0.07
7		Stem^c^	0.76 ± 0.15	0.71 ± 0.03
8		Leave^a^	15.56 ± 0.33	13.32 ± 0.16
9		Fruit^a^	12.05 ± 0.52	9.70 ± 0.10
10		Root^c^	0.93 ± 0.71	0.80 ± 0.26
11	Soxhlet^C^	Seed^e^	0.70 ± 0.03	0.07 ± 0.08
12		Stem^c^	11.37 ± 0.15	9.99 ± 0.10
13		Leave^a^	34.20 ± 1.05	41.40 ± 0.56
14		Fruit^b^	19.43 ± 0.92	16.59 ± 0.68
15		Root^d^	2.52 ± 0.86	1.87 ± 0.49

Explanations as given in Table 1

The brine shrimp lethality assay results are shown in Table 3. In case of shaking, the LC_50_ values were recorded of 0.40, 0.22, 1.49, 0.22, 3.71 concentrations (µg/mL) for seeds, stem, leaves, fruit and root, respectively, whereas seeds, stem, leaves, fruits and roots extracted by sonication method revealed the LC_50_ values of 9.92, 34.24, 2.12, 1.59, 60.92 (µg/mL), respectively and these values were 4.26, 0.72, 0.67, 8.62 and 33.60 (µg/mL) in case of Soxhlet extraction method. The LC_90_ values were found in the range of 345.42–1695.81 (µg/mL) (shaking), 660.50–14,794.40 (µg/mL) (sonication) and 641.62–15,047.80 (µg/mL) (Soxhlet). In case of brine shrimp assays, the plant different parts showed variable cytotoxicity level and extraction methods also affected the cytotoxicity level significantly. Overall, Soxhlet extracted samples showed higher cytotoxicity followed by sonication and shaking methods.

**Table 3:** Brine shrimp lethality assay of *Ricinus communis* different parts, extracted by different extraction methods

S. No.	Extraction	*R. communis*	LC_50_ (µg/mL)	95% confidence interval	LC_90_ (µg/mL)	95% confidence interval
1	Shaking	Seed	0.40	0.00–11.159	1695.81	239.99–5.92 × 10^10^
2		Stem	0.22	0.00–8.733	1405.67	177.77–2.69 × 10^24^
3		Leave	1.49	0.000119–12.838	345.42	81.46–5153.02
4		Fruit	0.22	0.00–8.733	1405.67	177.76–2.69 × 10^24^
5		Root	3.71	0.0099–20.963	446.66	130.65–4602.01
1	Sonication	Seed	9.92	0.0000033–75.920	13,212.00	1518.45–2.12 × 10^11^
2		Stem	34.24	0.0726049–164.453	14,794.40	2090.24–1908
3		Leave	2.12	0.00–29.647	5763.11	732.19–1.34 × 10^13^
4		Fruit	1.59	0.0000106–15.775	660.50	150.63–36,473.40
5		Root	60.92	4.793–186.931	4166.00	1214.41–95,587.90
1	Soxhlet	Seed	4.26	0.004573–26.076	786.29	217.29–17,185.70
2		Stem	0.72	0.00–13.359	1330.28	225.007–22,804
3		Leave	0.67	0.00–11.030	641.62	117.380–2756
4		Fruit	8.62	0.00243–54.525	3924.43	776.687–2411
5		Root	33.60	0.0438–162.661	15,047.80	2051.87–51,572

*Ricinus communis* methanolic extracts mutagenic results are shown in Table 4. In case of shaking extraction method, the TA98 revertant colonies were 2278, 2356, 2018, 2593 and 2628 (revertant colonies) for 50 µg extract/plate of seeds, stem, leaves, fruits and roots, respectively, whereas, 2139, 2072, 1975, 2471 and 2318 revertant colonies were recorded in case of sonication and for Soxhlet 1862, 1939, 2183, 2028 and 2319 revertant colonies were observed in response of seeds, stem, leaves, fruits and roots, respectively. TA100 strain showed a similar mutagenicity trend based on extraction methods and plant parts, however, the colonies reversion in case of TA100 were slightly higher than TA98 strain. In comparison to control, *R. communis* plant showed mutagenic nature. Regarding toxicity, there is lack of reports investigating the cytotoxicity and mutagenicity of *R. communis* using hemolytic, brine shrimp and Ames tests. However, these bioassays found to be short-term assays to evaluate the toxicity of extracts. These findings are in line with previous studies (Table 5), in which toxicity of this plant has also been reported in different models i.e., abrin and ricin (in *R. communis* extracts) reported to toxic by studying to SH- and S–S groups [75]. In another study, *R. communis* toxicosis in a sheep flock was studied and *R. communis* showed intoxication, in which most of the animals showed profuse watery diarrhoea, dehydration, weakness, salivation, mydriasis, teeth grinding, hypothermia and recumbency. High haematocrit, creatinine, high concentration of serum BUN and phosphorus and high activity of serum CK and AST were also observed along with cardiac haemorrhage, severe gastroenteritis, necrosis and acute tubular necrosis in kidneys and hepatic necrosis [76]. Antifeedant and toxic effects of leaf extracts of *R. communis* were also studied and results revealed that the extract had moderate effects towards these pests and author suggested the use of plant extract as a potential source of bioactive compounds for crop protectant against pest [77]. Antidiabetic activity of ethanolic extract of roots of *R. communis* also studied and 500 mg/kg BW showed promising efficiency in lowering the fasting blood glucose [78]. In view of results of the present investigation and reported studies, it can be concluded that *R. communis* is a potential source of bioactive compounds and could be used for the development of drugs for the treatment of various ailments.

**Table 4:** Mutagenicity of *Ricinus communis* different parts extracts, extracted by different extraction methods, tested by TA98 and TA100 strains of *Salmonella typhimurium*

S. No.	Methods	Plant part	Revertant colonies (mean ± SD)	
			TA_98_	TA_100_
1	Shaking	Seed	2278 ± 65	2773 ± 92
2		Stem	2356 ± 53	3056 ± 172
3		Leave	2018 ± 49	2939 ± 169
4		Fruit	2593 ± 128	3263 ± 174
5		Root	2628 ± 79	2562 ± 137
6	Sonication	Seed	2139 ± 130	3461 ± 147
7		Stem	2072 ± 219	3392 ± 67
8		Leave	1975 ± 67	3172 ± 119
9		Fruit	2471 ± 133	2938 ± 87
10		Root	2318 ± 104	2837 ± 234
11	Soxhlet	Seed	1862 ± 53	2978 ± 135
12		Stem	1939 ± 117	3038 ± 248
13		Leave	2183 ± 143	3365 ± 94
14		Fruit	2028 ± 138	3269 ± 182
15		Root	2319 ± 93	2957 ± 149
16	Positive control	0.25 (µg/plate)	3267 ± 278	
		0.5 (µg/plate)		4720 ± 346

Negative (solvent) control; *DMSO* dimethyl sulphoxide, *PC* positive control: for TA98, TN (0.25 µg/plate); for TA100, NQNO (0.5 µg/plate); Values are insignificant (*P* < 0.05) among plant parts and extraction methods, and significantly different (*P* < 0.05) form positive control

**Table 5:** Toxicities/activities reported for *Ricinus communis* plant

S. No.	Plant	Toxicities	References
1	*Ricinus communis*	Toxicity against SV40-transformed 3T3 fibroblasts	[79]
2	*Ricinus communis*	Toxicity against brown Hisex chicks fed diets containing 0.5% *R. communis* seed	[80]
3	*Ricinus communis*	Toxicity against leaf-cutting ant Atta sexdens rubropilosa Forel	[81]
4	*Ricinus communis*	Toxicity against nests of Atta sexdens rubropilosa	[82]
5	*Ricinus communis*	Toxicosis in a sheep flock	[76]
6	*Ricinus communis*	Toxicity against pests	[77]
7	*Ricinus communis*	Antidiabetic activity	[78]
8	*Ricinus communis*	Anti-tumor activity	[83]

### DNA protection

**Fig. 1:**
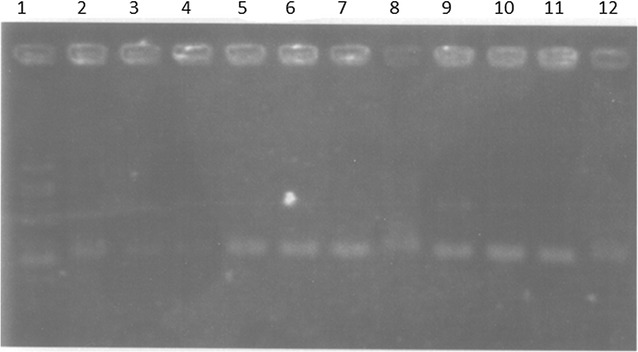
DNA damage/protection effect of methanol extract of *Ricinus communis* exposed to H_2_O_2_ and UV induced oxidative damage on pBR^322^. Lane 1 = 1 Kb DNA ladder: lane 2 = Plasmid pBR^322^ DNA without treatment (super coiled); lane 3 = Plasmid pBR^322^ DNA treated with H_2_O_2_ (open circular or damaged), lane 4 = Plasmid pBR^322^ DNA; treated with H_2_O_2_ + UV (open circular or damaged); lane 5 = Plasmid pBR^322^ DNA treated seed extract by shaking method + H_2_O_2_; lane 6 = Plasmid pBR^322^ DNA treated with stem extract by shaking method + H_2_O_2_; lane 7 = Plasmid pBR^322^ DNA treated with leaves extract by shaking method + H_2_O_2_; lane 8 = Plasmid pBR^322^ DNA treated with fruits extract by shaking method + H_2_O_2_; lane 9 = Plasmid pBR^322^ DNA treated with seeds extract by shaking method + H_2_O_2_ + UV light); lane 10 = Plasmid pBR^322^ DNA treated with stems extract by shaking method + H_2_O_2_ + UV); lane 11 = Plasmid pBR^322^ DNA treated with leaves extract by shaking method + H_2_O_2_ + UV); lane 12 = Plasmid pBR^322^ DNA treated with fruits extract by shaking method + H_2_O_2_ + UV)

DNA protection assay was performed by inducing DNA damage by UV light and H_2_O_2_. The NDA damage caused by H_2_O_2_ and UV radiation and extracts protection efficiency was studied using Plasmid pBR^322^. In DNA damage, H_2_O_2_ generates OH· as shown in Eq. 4, which are responsible for DNA breakage through oxidative reaction (Eq. 5) [84, 85]. The Plasmid pBR^322^ DNA damage and protective results are shown in Fig. 1. The Plasmid pBR^322^ DNA ladder band is clear (lane 1), whereas Plasmid pBR^322^ DNA treated with H_2_O_2_ revealed that DNA damage was damaged (lane 3). The UV light and H_2_O_2_ in combination also induced Plasmid pBR^322^ DNA (lane 4). The Plasmid pBR^322^ DNA treated with *R. communis* extracts (extracted by different methods) in the presence of H_2_O_2_ + UV results are shown in lanes 5–12. Results revealed that H_2_O_2_ + UV induced Plasmid pBR^322^ DNA damage was protected. The H_2_O_2_ + UV treated DNA converted the Plasmid pBR^322^ into open circular form, whereas upon treatment with the extract regained the native form of Plasmid pBR^322^ DNA, which revealed the *R. communis* extracts protected DNA from the OH· induced damage. As it is well known that OH· is a strong oxidative agent and can damage the DNA by oxidation process, which indicates that free radical induced DNA damage cab be protected using *R. communis* extract. Since Plasmid DNA is damaged by OH· radical by free radical-induced chain reaction mechanism and OH· react with nitrogenous bases producing base free radical and other radicals. The base radical in turn reacts with the sugar moiety causing breakage of sugar phosphate backbone of nucleic acid resulting in strand break [85, 86]. Previous studies also supported these results that plant extract can protect DNA damage, i.e., *D. bipinnata* extract prevented the oxidative damage to DNA in the presence of a DNA damaging agent (Fenton’s reagent) at a concentration of 50 μg/mL. Also, the presence of extract protected yeast cells in a dose-dependent manner from DNA damaging agent [85]. Recently, the DNA damage inhibition potential of a methanolic extract of *C. carandas* leaves were also studied [87]. It was reported that extract showed significant H_2_O_2_ scavenging activity (median inhibitory concentration, 84.03 μg/mL) and completely protected pBR^322^ Plasmid DNA from free radical-mediated oxidative stress. Authors correlated the DNA damage inhibition with high content of phenolic compounds in *C. carandas* extracts. In another study, the free-radical scavenging properties and potential to prevent DNA damage of 56 extracts from 14 medicinal plants were studied. The extracts protected DNA against photolyzed H_2_O_2_-induced oxidative damage by all plant extracts [88]. So far, results revealed that the *R. communis* extract has ability to protect DNA damage and present study provides roadmap for identification and isolation of bioactive compounds and possible use to manage the free radical induced diseases.

4 $${\text{H}}_{2} {\text{O}}_{2} {\text{--}}^{ \wedge } {\text{-}}^{ \wedge } {\text{--}}{>} 2{\text{OH}} \cdot$$

5 $${\text{RH}} + {\text{OH}} \cdot \to {\text{H}}_{ 2} {\text{O}}/{\text{N}} - {\text{bases }} + {\text{R}} \cdot \to {\text{Oxidative by-products}}$$

## Conclusions

Cytotoxicity, mutagenicity, antioxidant as well as DNA protective efficiency of *R. communis* (seeds, stem, leaves, fruit and root) methanolic extracts were evaluated. Extracts showed variable antioxidant activity among plant parts and extraction methods. The *R. communis* also protected Plasmid pBR^322^ DNA from H_2_O_2_ and UV damage. Bioassays (Hemolytic, brine shrimp and Ames test) revealed that the *R. communis* methanolic extracts have compounds responsible for mild to moderate to moderate toxicity. *R. communis* may be a potential source of compounds for the development of new medicine and future studies will be focused on the identification of compounds responsible for bioactivity.
